# Boron neutron capture therapy with bevacizumab may prolong the survival of recurrent malignant glioma patients: four cases

**DOI:** 10.1186/1748-717X-9-6

**Published:** 2014-01-06

**Authors:** Shin-Ichi Miyatake, Shinji Kawabata, Ryo Hiramatsu, Motomasa Furuse, Toshihiko Kuroiwa, Minoru Suzuki

**Affiliations:** 1 Department of Neurosurgery, Osaka Medical College, Osaka, Japan; 2 Radiation Oncology and Particle Radiation Oncology Research Center, Research Reactor Institute, Kyoto University, Kyoto, Japan

**Keywords:** Bevacizumab, Boron neutron capture therapy, Recurrent malignant glioma

## Abstract

**Background and importance:**

Recurrent malignant gliomas (RMGs) are very difficult to control, and no standard treatments have been established for them. We performed boron neutron capture therapy (BNCT) for patients with RMG. BNCT enables high-dose particle radiation to be applied selectively to tumor cells. However, RMG cases generally receive nearly 60 Gy X-ray irradiation prior to re-irradiation by BNCT. Therefore, even with tumor-selective particle radiation BNCT, radiation necrosis in the brain and symptomatic pseudoprogression may develop. In four of our recent patients with RMG after BNCT, we applied the anti-VEGF antibody bevacizumab to treat two pathological entities. This approach appeared to prolong survival. Here we present the case reports of these four consecutive patients with RMG and discuss the novel use of bevacizumab in this context.

**Clinical presentation:**

Four patients with RMGs were treated with BNCT at our institutes. Upon the referral for BNCT, they were assessed as belonging to the recursive partitioning analysis (RPA) class 3 (n = 3 patients) or RPA class 4 (n = 1 patient) (the RPA classification for RMG was advocated by Carson et al. in 2007). The estimated median survival times for RPA classes 3 and 4 were 3.8 and 10.8 months, respectively, after some treatment at the recurrence. We applied BNCT for these four patients and administered bevacizumab when the lesions were considered radiation necrosis or symptomatic pseudoprogression. The class 3 patients survived after the BNCT for 14, 16.5 and > 23 months, and the class 4 patient survived > 26 months, with favorable improvements in clinical symptoms.

**Conclusion:**

BNCT with the addition of bevacizumab for radiation necrosis or symptomatic pseudoprogression improved the clinical symptoms and prolonged the survival in RMG patients.

## Background

The prognosis of recurrent malignant gliomas (RMGs) is poor, and no standard treatment has been established [1]. Since 2002 at our institute, we have been applying a form of tumor-selective particle radiation, boron neutron capture therapy (BNCT), for RMGs and observed favorable survival outcomes [2,3]. BNCT is a biochemically targeted radiotherapy based on the nuclear capture and fission reactions that occur when non-radioactive boron-10, which is a constituent of natural elemental boron, is irradiated with low-energy thermal neutrons to yield high-linear-energy transfer alpha particles and recoiling lithium-7 nuclei. These particles are released within a very short range such as 9 μm, and therefore the cytotoxic effects are confined within boron-10-containing cells [4].

Boron-10-containing compounds can be accumulated selectively in tumor cells by several mechanisms. For example, boronophenylalanine (BPA) is selectively and preferentially accumulated in tumor cells via the augmented metabolism of amino acids compared to normal cells. Even with this novel and selective particle radiation technique, radiation damage — chiefly radiation necrosis (RN) and symptomatic pseudoprogression (psPD) — often occurs [5,6]. The radiation damage is especially likely in RMG cases, because full-dose X-ray treatment (XRT) is generally part of the treatment history in such cases.

Bevacizumab (BV), an anti-vascular endothelial growth factor (VEGF) antibody, has recently been used for the treatment of symptomatic RN [7,8]. Based on our analysis of human RN surgical specimens, we previously demonstrated that the edema in RN is caused by the overexpression of VEGF in reactive astrocytes [9]. Following this determination, we used BV in an attempt to control the symptomatic RN and the symptomatic psPD encountered after BNCT for RMGs [5,7]. Here we present a case series report of our last four consecutive cases of RMG treated with BNCT and BV, with >18-month observation periods. All four patients had RMGs after primary treatment with XRT and chemotherapy consisting chiefly of temozolomide (TMZ). The patients’ profiles and survival data are listed in Table 1. Three of the patients were classified as recursive partitioning analysis (RPA) (advocated by Carson et al. in 2007 [1]) class 3 and one was classified as RPA class 4.

**Table 1:** The background of the four patients with recurrent malignant glioma (RMG)

**Case No.**	**Age**	**Sex**	**Hist.**	**RPA class**	**Irradiated dose (Gy-Eq)**			**BV cycles**	**PsPD or RN**	**Survival (Months from BNCT)**
					**Brain (Max)**	**Tumor (Max)**	**Tumor (Mini)**	**(Months from BNCT)**		
1	43	M	AA	3	11.4	118	36.1	3 (11 M)	RN	23 M, alive
2	41	M	GBM	4	12.1	88.5	36.6	4 (14 M)	RN	26 M, alive
3	60	M	AA	3	10.8	110	82.3	6 (4 M)	PsPD	16.5 M
4	34	F	AOA	3	11.5	71.6	30.1	6 (2 M)	PsPD	14 M

Hist, histology; RPA, recursive portioning analysis; BV, Bevacizumab; PsPD, pseudoprogression; RN, radiation necrosis; BNCT, boron neutron capture therapy.

## Case presentation

### Case 1

**Figure 1:**
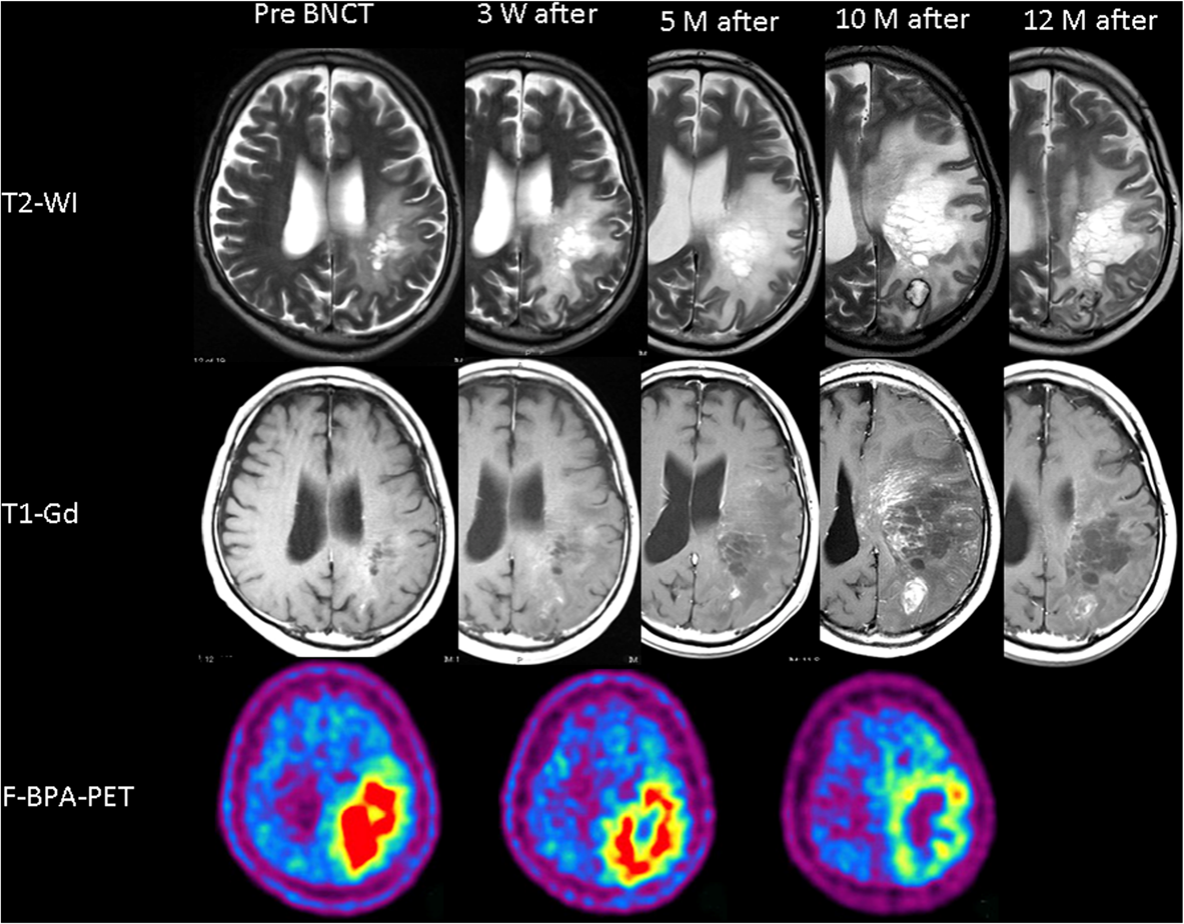
**Sequential change of T2-weighted MRI (upper column), Gd-enhanced T1-weighted MRI (middle column) and F-BPA-PET (lower column) of Case 1, a 44-year-old male.** The timing of the MRIs is depicted above the MRIs. F-BPA-PET images were taken just before the BNCT and at 1 month and 10 months after the BNCT. These PET images show the gradual decrease of the tracer uptake as a promising effect of the BNCT. BV was started 10 months after the BNCT, and the MRI showed marked improvement of both perifocal edema and contrast enhancements by BV treatment.

A 44-year-old male’s craniotomy showed anaplastic astrocytoma. He received standard chemoradiotherapy (XRT 60 Gy with TMZ). Unfortunately the lesion recurred with aggravation of aphasia and right hemiparesis, which forced him to retire from his job. The Karnofsky performance status (KPS) was 70%, and he was classified as RPA class 3. The patient was then referred to our institute for BNCT. Upon referral, MRI showed a slightly enhanced lesion with mild perifocal edema (Figure 1). A simultaneous fluorine-18-labeled BPA positron emission tomography (F-BPA-PET) image showed marked tracer uptake in the left parietofrontal region (Figure 1), with a 6.0 lesion/normal (L/N) brain ratio of the tracer, indicating that the lesion was a highly malignant tumor. BNCT was applied for this patient according to our recent protocol for RMGs and meningiomas [10]. Briefly, only BPA was administered over a 2-hr period (200 mg/kg/hr) just prior to and during the neutron irradiation (100 mg/kg/hr). The neutron irradiation time was decided based on a simulation not to exceed 12.0 Gy-Eq (Gray-equivalent) for the peak brain dose. The 10-B concentration in the blood during the neutron irradiation was 23.0 parts per million (ppm). By BNCT, the maximum brain dose, maximum tumor dose, and minimum tumor dose were estimated as 11.4, 118, and 36.1 Gy-Eq, respectively. Here, "Gy-Eq" corresponds to the biologically equivalent X-ray dose that would have equivalent effects on tumors and on the normal brain. The dose estimation was performed by the measurement of blood boron concentration and F-BPA-PET data prior to neutron irradiation as described elsewhere [2,6,10].

After the BNCT, an MRI showed gradual enlargement of both perifocal edema and contrast enhancement, whereas sequential F-BPA-PET showed a favorable decrease of tracer uptake (Figure 1, lower panel). F-BPA-PET was originally developed to estimate the absorbed dose in BNCT, as described above [2,11,12]. The background uptake of the tracer F-BPA is very low compared to that of fluorodeoxy-glucose and even compared to that of methionine as a tracer. Thereafter, RN and psPD have been differentially diagnosed from tumor progression by F-BPA-PET [6,13]. Ten months after the BNCT, the patient’s KPS worsened to 60%, and so we administered BV 5 mg/kg biweekly, three times. Just prior to the BV administration, F-BPA-PET showed a more decreased L/N ratio, which indicated that the aggravation shown by MRI was RN and not a recurrence of the tumor. After the BV treatment, MRI showed improvement of the perilesional edema and a decrease in contrast enhancement. The BV treatment stabilized the patient’s symptoms for 6 months but then his symptoms recurred, prompting us to perform a re-challenge with BV another three times. The patient is now stable and doing well, 23 months after the BNCT (Table 1).

### Case 2

**Figure 2:**
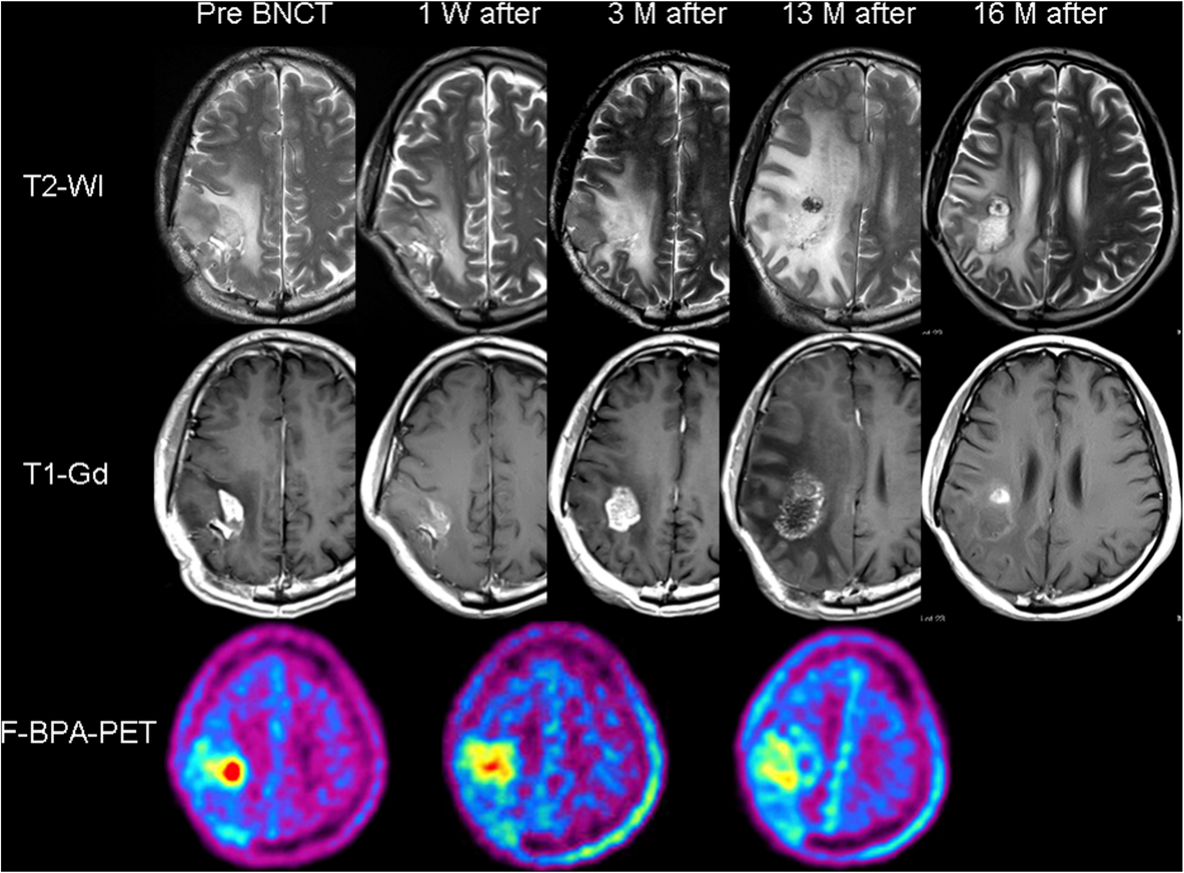
**Sequential change of T2-weighted MRI (upper column), Gd-enhanced T1-weighted MRI (middle column) and F-BPA-PET (lower column) of Case 2, a 41-year-old man.** The timing of the MRI is depicted above the MRI. F-BPA-PET images were taken just before the BNCT, 1 month after and 12 months after the BNCT. These PET images show the gradual decrease of the tracer uptake as a promising effect of BNCT. BV was started 13 months after the BNCT, and an MRI showed a marked positive effect of the BV treatment on the perifocal edema and contrast enhancements.

A 41-year-old man underwent surgery for his right parietal glioblastoma (GBM) with subtotal excision. Standard treatment with XRT and TMZ was performed, but the tumor recurred 5 months after the surgery. Upon referral for BNCT, the patient’s KPS was assessed as 90% and he was classified as RPA class 4. MRI showed a definitively enhanced lesion with moderate perifocal edema (Figure 2). A simultaneous F-BPA-PET image showed marked tracer uptake in the right parietal region with a 3.8 L/N ratio of the tracer, indicating that the lesion was a recurrent malignant tumor and not psPD (Figure 2, lower panel). He was treated with BNCT, with the same protocol as Case 1. The boron-10 concentration in the blood during the neutron irradiation was 30.2 ppm. By BNCT, the maximum brain dose, maximum tumor dose, and minimum tumor dose were estimated as 12.1, 88.5, and 36.6 Gy-Eq, respectively. One week after the BNCT, a contrast-enhanced T1-weighted MRI showed a marked shrinkage of the mass, and that at 3 months later showed slight enlargement of the enhanced lesion, which was presumed to be psPD. Periodic MRIs showed gradual enlargement of both the enhanced lesion and perifocal edema, whereas F-BPA-PET showed a gradual decrease of the tracer uptake. The final L/N ratio, 1 year after BNCT, was 2.3. This L/N ratio and the MRI 13 months after the BNCT suggested that the lesion was RN.

The patient was not able to continue his work as a cook, and we decided to begin intravenous BV treatment biweekly (5 mg/kg). After four treatments, MRI showed marked improvement in the perifocal edema and left hemiparesis. The patient is now doing well and has resumed his work as a cook, 26 months after the BNCT, without tumor progression or recurrence of the RN.

### Case 3

A 56-year-old male experienced speech disturbance and mild right hemiparesis. First he received a craniotomy with a diagnosis of gemistocytic astrocytoma, followed by fractionated XRT (total 50 Gy) and repetitive chemotherapy with nitrosourea. Three years later, a recurrent lesion appeared with Gd enhancement on MRI. Re-craniotomy revealed GBM histologically. After surgery, the enhanced lesion gradually grew and the patient’s sensory aphasia worsened despite the repeated administration of TMZ. He was referred to our institute for BNCT. Upon his referral, he was assessed as RPA class 3. The boron-10 concentration in the blood during the neutron irradiation was 30.0 ppm. Using BNCT, the maximum brain dose, maximum tumor dose, and minimum tumor dose were estimated as 10.8, 110, and 82.3 Gy-Eq, respectively, as shown in Table 1. His right hemiparesis and aphasia gradually worsened after the BNCT, even with an escalating dose of corticosteroids. Four months after the BNCT, a follow-up MRI and F-BPA-PET suggested that the lesion was symptomatic psPD, not tumor progression. The patient was successfully treated with BV, as we recently reported, along with the periodic changes of the neuroimages and the detailed clinical course [5]. We lost this patient to local tumor progression 16.5 months after the BNCT.

### Case 4

A 27-year-old female manifested left hemiparesis. A right frontal enhanced mass was removed gross/totally, and the histological diagnosis was anaplastic oligo-astrocytoma. She received fractionated XRT (total 72 Gy) and repetitive chemotherapy with nitrosourea. The lesion recurred and re-craniotomy was performed 4 years later, with the same pathological diagnosis. This was followed by TMZ chemotherapy. Unfortunately, a recurrence was confirmed by MRI and she was referred to us for BNCT. The boron-10 concentration in the blood during the neutron irradiation was 21.4 ppm. By BNCT, the maximum brain dose, maximum tumor dose, and minimum tumor dose were 11.5, 71.6, and 30.1 Gy-Eq, respectively (Table 1). After the BNCT, her hemiparesis gradually became aggravated despite an increased dose of corticosteroids. MRI taken 2 months after the BNCT showed an enlarged enhanced lesion with increased perilesional edema. We judged this aggravation as symptomatic psPD. We started BV treatment for her. The patient was bedridden just prior to the BV treatment, but after two BV treatments her hemiparesis improved markedly and she could walk. Her neuroimages and clinical symptoms showed marked improvement, as we reported previously [5]. Unfortunately we lost her because of tumor extension to the opposite hemisphere 14 months after the BNCT.

The neuroimages, including F-BPA-PET scans of Cases 3 and 4, were published elsewhere [5] and thus are not included in this brief report.

## Discussion

In comparison with many Phase I and II trials for RMG [1], BNCT showed a marked survival benefit for RMG in our previous study, in which BV was not used [3]. Briefly, BNCT resulted in median survival times (MSTs) (months and 95% confidence intervals) as follows: for all RPA classes (Classes 1–7), 10.8 (7.3–12.8) (n = 22), and in the poor-prognosis group (RPA class 3 + 7), 9.1(4.4–11.0) (n = 11). In a meta-analysis reported in the Journal of Clinical Oncology [1], the MSTs in all RPA classes and in the poor-prognosis group (RPA class 3 + 7) were 7.0 (6.2–8.0) (n = 310) and 4.4 (3.6–5.4) (n = 129), respectively. These data showed the superiority of BNCT for RMGs, especially in poor-prognosis groups. In comparison, our previous data showed MSTs of RPA class 3 and 4 as 7.3 and 12.0 months, respectively, although the number of the patients was quite limited: 4 cases in class 3 and 3 cases in class 4 [3].

In our recent patients undergoing BNCT for RMGs, we have begun to treat RN or symptomatic psPD aggressively by administering BV. We applied intravenous BV treatment for four recent RMG patients treated with BNCT at our institute and in whom we encountered RN or symptomatic psPD; these cases are reported here. Three of these four patients were classified as RPA class 3 and one as class 4 (Table 1). The estimated survival time of class 3 patients is 3.8 months and that of class 4 patients is 10.8 months [1]. Our three class 3 patients survived for 14, 16.5, and > 23 months, and the class 4 patient has survived for over 26 months.

At a glance, BNCT with BV seemed to prolong the survival of RMGs strikingly in comparison not only with Carson’s data set but also with our previous BNCT data. Although of course no definitive conclusion can be drawn from such a small number of cases.

In our limited experience, there is no obvious histological difference between RN and psPD [6]. The center part of each pathology is characterized as histological necrosis, and marked angiogenesis is observed in the boundary of the necrotic core and normal brain tissue [9]. Clinically, most psPD occurs at a relatively early stage after intensive treatments and is self-limiting without severe sequelae [14]. In most cases, psPD improves over time without intensive treatments. On the other hand, RN often shows severe symptoms and occurs at least a half year after radiotherapy. Thereafter, symptomatic psPD is especially difficult to distinguish from RN. In Table 1, we distinguish them only from the duration of the symptomatic onset after BNCT.

We have described herein the use of BV for RN or psPD after BNCT. BV was approved for the treatment of RMGs as an anticancer agent [15,16], and several trials of re-irradiation using XRT or hypo-fractionated stereotactic radiotherapy in combination with BV just before radiotherapy for RMGs have recently been conducted, with favorable preliminary safety and response results [17-19]. The authors of those reports described the role of BV not only as an anticancer agent but also for normalizing the perfusion pressure and oxygenation effects during irradiation. BV may also prevent RN and symptomatic psPD after re-irradiation.

We are now planning a prospective clinical trial of BNCT using BV immediately after neutron irradiation for RMG patients with poor prognosis (class 3 + 7). We are also conducting a clinical trial of BNCT for RMGs using a small accelerator in-hospital, instead of an atomic reactor. We hope to determine whether accelerator-based BNCT with BV could be used as a standard treatment for RMGs.

## Conclusion

BNCT with the addition of BV for radiation necrosis or symptomatic pseudoprogression improved the clinical symptoms and might prolong the survival of RMG patients.

### Consent

Written informed consent was obtained from the patient for the publication of this report and any accompanying images.

## Abbreviations

BNCT: Boron neutron capture therapy; BPA: Boronophenylalanine; BV: Bevacizumab; GBM: Glioblastoma; Gy-Eq: Gray-equivalent; KPS: Karnofsky performance status; L/N: Lesion/normal; PET: Positron emission tomography; ppm: parts per million; psPD: pseudoprogression; RMG: Recurrent malignant gliomas; RN: Radiation necrosis; RPA: Recursive portioning analysis; TMZ: Temozolomide; XRT: X-ray treatment.

## Competing interests

There is no conflict of interest to disclose for any of the authors.

## Authors’ contributions

S-IM conceived of the study and participated in the follow-up of patients. SK, RH, and MS applied BNCT in the atomic reactor. MF followed the patients with bevacizumab. TK selected the patients for BNCT. All authors read and approved the final manuscript.
